# Capacity Building for Dengue Prevention: A Comprehensive Training Approach in Two Medical Colleges in Puducherry, India

**DOI:** 10.7759/cureus.77042

**Published:** 2025-01-06

**Authors:** Arya Rahul, Rajendran Dhanalakshmi, Srikanth Srirama, Shriram A Nagarajan, Arunachalam D Dhakshinamoorthy, Ashwyn Nelson, Prem Anand, Subalakshmi Subramaniyan, Vasanthakumari Ramadass, Manju Rahi

**Affiliations:** 1 Epidemiology and Public Health, Indian Council of Medical Research (ICMR) - Vector Control Research Centre, Puducherry, IND; 2 Epidemiology and Operational Research, Indian Council of Medical Research (ICMR) - Vector Control Research Centre, Puducherry, IND; 3 Vector Biology and Control, Indian Council of Medical Research (ICMR) - Vector Control Research Centre, Puducherry, IND; 4 Community Medicine, Aarupadai Veedu Medical College and Hospital, Puducherry, IND; 5 General Surgery, Aarupadai Veedu Medical College and Hospital, Puducherry, IND; 6 Community Medicine, Sri Manakula Vinayagar Medical College and Hospital, Puducherry, IND; 7 Infectious Diseases, Directorate of Health and Family Welfare Services, Puducherry, IND

**Keywords:** capacity building, competency development, dengue source reduction, health capacity building, health education, multidisciplinary teams, risk communication, stakeholder engagement, teaching strategies, vector control

## Abstract

Background

Dengue, a neglected tropical disease, is becoming a more significant global health concern due to factors such as unplanned urbanization, poor sanitation, and improper protective measures. Dengue transmission is intricately linked to the knowledge, perspectives, and practices, and sustainable vector control activities in preventing dengue transmission. We aimed to develop a training plan and implementation strategy for Dengue Source Reduction (DSR) activities for building capacity in medical colleges in Puducherry, India, to control vectors and prevent dengue.

Methods

This quasi-experimental study involved two medical colleges. Key stakeholders were identified, and resources available for the training program were mapped. The training sessions were interactive, incorporating lectures, demonstrations, discussions, and hands-on training on identifying and removing vector breeding habitats. Sixty multidisciplinary teams (MDTs) of 5-6 members were created, each assigned a zone within the college campus. Each team had a leader responsible for coordinating activities, communication, and reporting. Monitoring and evaluation included pre- and post-training surveys, assessments of capacity before and after the training, and identification of collected mosquito larvae.

Results

The training program, with a focus on interactive learning and robust monitoring, led to significant improvements in knowledge, perception, and capacity. Stakeholder engagement led to the development of the "Detect, Destroy, and Document (3D)" approach for monitoring and evaluation. Participants showed increased knowledge scores (6.79±1.16 to 8.07±0.89 out of 9, paired t-test p<0.001), heightened risk perception (16.7% to 55%, McNemar's test p<0.001), and a stronger understanding (63.3% to 86.7%, McNemar's test p=0.0005) of the importance of weekly DSR activities. The knowledge scores of breeding sites, symptoms, and preventive measures improved significantly after the intervention. Furthermore, participants demonstrated the ability to identify common *Aedes *breeding sites and mosquito larvae and even conducted a follow-up DSR activity independently. The number of containers identified improved during the follow-up. On emergence in the laboratory, all the larvae collected during the independent follow-up belonged to *Aedes* mosquitoes.

Conclusions

Interactive hands-on capacity-building programs in medical colleges can be a promising approach to reducing dengue transmission risks. Integrating vector control training into the medical curriculum and sustaining motivated and trained MDTs within medical colleges can have a broader impact on community health.

## Introduction

Dengue, a mosquito-borne viral disease, is classified as a neglected tropical disease, with a rising global burden. In 2023, there were five million reported cases of dengue in the world with 5,000 dengue-related deaths and 289,235 cases in India with 485 deaths [1,2]. The *Aedes *mosquito, specifically infected females, transmits the virus through bites. These mosquitoes breed in both indoor and outdoor water collections, making them prevalent in urban environments [3]. Unplanned urbanization creates ideal conditions for *Aedes *mosquitoes, increasing dengue transmission due to factors such as population density, travel patterns, rainfall, and water storage practices [4]. Public knowledge, perception, and practices regarding dengue, along with sustainable community-based vector control, are crucial for controlling dengue.

Puducherry, a Union Territory in India, experiences frequent dengue outbreaks probably due to high transmission rates [2]. As per the 2011 Census, Puducherry, with a 294 km² area, has a population of 950,289 with a population density of 3,232 per km². Limited resources make controlling and preventing these outbreaks a significant challenge. Therefore, there is a critical need to formulate sustainable and cost-effective dengue control interventions. There are two government and eight private undergraduate medical colleges in Puducherry. Medical colleges, with their dedicated healthcare workforce and established links to urban and rural health and wellness centers, in the areas from where most of their patients' families live and where most of their public health initiatives are carried out, are well-positioned to play a key role in dengue control. Engaging medical colleges offers several advantages. Leveraging their unique position within communities, medical colleges can become hubs for comprehensive dengue control. By implementing effective anti-dengue measures on their campuses, they can not only reduce the institutional risk of outbreaks but also serve as a model for surrounding communities.

Medical college campuses occupy a large area within the communities and may inadvertently act as a breeding ground for dengue vectors such as *Aedes aegypti *and *Aedes albopictus*. Furthermore, the presence of dengue-positive patients within the campus, coupled with the existence of vectors, amplifies the risk of disease transmission. Consequently, medical colleges are well suited to lead dengue control action plans, seamlessly extending their impact to the broader community. Leveraging their educational expertise, they can spearhead awareness campaigns and knowledge transfer initiatives, empowering residents to identify and eliminate potential mosquito breeding sites within their own homes and neighborhoods. This comprehensive approach has the potential to significantly reduce the overall risk of dengue transmission.

The study aims to engage with stakeholders in two medical colleges in Puducherry to develop a comprehensive training plan and implementation strategy framework for Dengue Source Reduction (DSR) activities, strengthening dengue prevention efforts at the medical college level and empowering them to reduce mosquito breeding sites. Additionally, it seeks to form a multidisciplinary team (MDT) and assess the change in their knowledge, perceptions, and practices related to dengue prevention and vector control after implementing the training plan.

## Materials and methods

**Figure 1:**
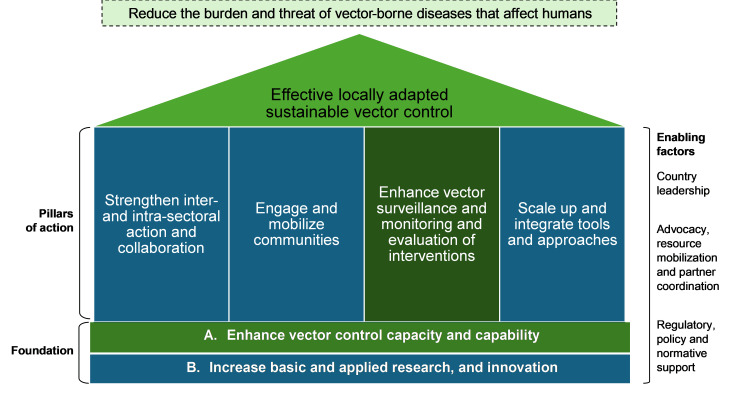
Global Vector Control Response framework GVCR: Global Vector Control Response Source: Recreated from the World Health Organization's GVCR document [5]

By focusing on Dengue Source Reduction (DSR) activities, medical colleges can become leaders in controlling dengue within their institutions and surrounding communities. The Global Vector Control Response (GVCR) framework of the World Health Organization (WHO) outlines a strategic approach for tackling vector-borne diseases. A pivotal element within this framework involves enhancing capacity and capabilities for effective vector control (Figure 1) [5]. This quasi-experimental study aimed to develop an implementation strategy for building capacity in medical colleges for vector control and dengue prevention and evaluate the training plan.

Training sites

Training was conducted at two medical colleges in Puducherry, India: Aarupadai Veedu Medical College and Hospital and Sri Manakula Vinayagar Medical College and Hospital. Both colleges are partnered with ICMR-Vector Control Research Centre for collaboration on research and capacity-building activities. They have 540- and 1,180-bed teaching hospitals attached, respectively, with clinics that see over 300 febrile cases monthly. While there have been general cleanliness drives and maintenance reviews, there have not been any regular annual targeted source reduction activities within the campuses.

Stakeholder engagement and resource mapping

The collaborating institutes were visited to identify key stakeholders and conduct a comprehensive resource mapping exercise. Detailed discussions were held with hospital administrators (medical superintendents and deputy medical superintendents) and department heads regarding the dengue control action plan. An activity plan/training program was formulated with a tailored "Detect, Destroy, and Document (3D)" approach based on insights from the stakeholder engagement sessions.

Formation of multidisciplinary teams (MDTs)

Sixty multidisciplinary teams, each comprising 5-6 members from diverse backgrounds, including faculty and students from medical, nursing, and other paramedical disciplines, and housekeeping staff were established. The campus was subdivided into 10-15 manageable zones, with each team assigned a specific zone for their DSR efforts. To ensure smooth operation, each team had a designated leader responsible for overseeing team dynamics, communication management, and report submission.

Interactive training for capacity building

Training sessions were designed by integrating three communication models to enhance participants' knowledge and skills in vector control: "transmission model", a traditional approach in which trainers deliver information directly to participants [6], "social learning", participants learn by observing the actions and experiences of others [7], and "experiential learning", participants actively participate in exercises and discussions, allowing for reflection and deeper understanding [8].

The MDTs received a comprehensive 30-minute training session delivered by trained investigators using the WHO Integrated Vector Management (IVM) training module [9]. The training covered key topics including dengue fever overview, characteristics of *Aedes* mosquitoes, their life cycle stages, common breeding sites found in hospitals and communities, and practical methods for reducing breeding sources (transmission model of communication). Live mosquito samples at various stages (eggs, larvae, and pupae) were used for demonstration during the training. This was carried along with small group discussions concurrently by the facilitators. The training and discussion sessions were held in both English and the local language (Tamil). This approach ensured clear communication, created an inclusive learning environment, and equipped the MDTs with the necessary skills for successful field activities (social learning model of communication). To enhance understanding and participation, the training transitioned to a hands-on component. Here, MDTs were guided by two trainers, including Master of Public Health Entomology (MPHE) students specializing in vector biology and control, to identify and eliminate potential mosquito breeding sites within their assigned zones (a mix of social and experiential learning models).

Monitoring and evaluation

A monitoring and reporting system was established to track progress and identify areas for improvement. Team leaders submitted detailed reports after each activity. These reports included photographs as evidence and documented the teams' activities, any challenges encountered during DSR efforts, and the outcomes achieved within their assigned zones. This monitoring plan aligned with the "Detect, Destroy, and Document (3D)" approach was developed in collaboration with stakeholders. This iterative process allowed for continuous monitoring and improvements in the DSR action plan. This plan was created along the principles of evaluation laid out by the Centre for Disease Control and Prevention (CDC) [10].

Evaluation measures

The reception of the training program was assessed through various methods.

Knowledge and Perception

Pre- and post-training evaluations measured changes in participants' knowledge and perception toward dengue using a pre-validated questionnaire adapted from Govindasamy et al. [11].

Capacity Assessment

Supervised baseline (immediately following training) and unsupervised follow-up evaluations (after 10 days) assessed participants' ability to identify and eliminate potential vector breeding sites within the college campuses.

Confirmation of Mosquito Species

Larval samples collected during the DSR activities were raised in the Centre's laboratory for taxonomic identification by the research team to confirm the presence of mosquito vectors.

Data were collected through assessments and entomological analysis following the Kirkpatrick model's four levels [12]. The evaluation included immediate post-training discussions to gauge the reaction (level 1), pre- and post-training knowledge assessments (level 2), follow-up activity and observations (level 3), and entomological measurements (level 4) to look for retention and independent performance.

Statistical methods

The pre-test and post-test data for cognitive evaluation were collected using anonymous Google Forms (Google, Inc., Mountain View, CA). The forms were linked using unique team codes on both pre-test and post-test forms. The knowledge components were individually scored, and a composite score was derived by adding them. Descriptive statistics were employed for continuous data, presented as mean ± standard deviation (SD). Paired t-test was used to assess the differences in continuous data between pre-test and post-test scores. Categorical data are presented as proportions, and McNemar's test was employed to evaluate the changes in proportions between the pre-test and post-test results. The data was analyzed using SPSS version 21 (IBM Corp., Armonk, NY). A probability (p) value < 0.05 was considered significant.

## Results

Stakeholder engagement

A meeting was held with administrators (dean of student affairs, medical superintendent, and assistant medical superintendents) and department heads (medicine, community medicine, microbiology, and housekeeping), after obtaining due permissions. A separate meeting was also held with the state Vector Borne Diseases Control Programme (NVBDCP) team. We engaged with 14 stakeholders (10 men, 71%) who were aged between 34 and 62 years, with an experience between six and 32 years.

There were no dedicated fever clinics. The Department of Internal Medicine managed cases of dengue fever as per guidelines. Probable cases underwent screening tests for dengue NS1 antigen and immunoglobulins (IgG and IgM). Positive reports were shared daily with the national Integrated Disease Surveillance Program (IDSP). The institutes also reported cases of dengue among campus residents. However, there were no targeted Dengue Source Reduction (DSR) activities, and no risk communication materials were available for staff and the community. The stakeholders also deliberated on the challenges of doing regular DSR activities. These challenges included people not being worried enough about dengue (lack of risk perception), not having enough trained staff, difficulty getting staff and students to help with cleanup activities, and scheduling conflicts with work or academic classes.

The discussion also focused on how the DSR activity can be implemented. The college team presented the campus layout and divided it into zones for DSR activity. Resource mapping was conducted to identify available resources, and the composition of multidisciplinary survey teams was determined accordingly. Special attention was given to involving students and staff from medical, nursing, and paramedical departments, along with housekeeping staff in each team.

**Figure 2:**
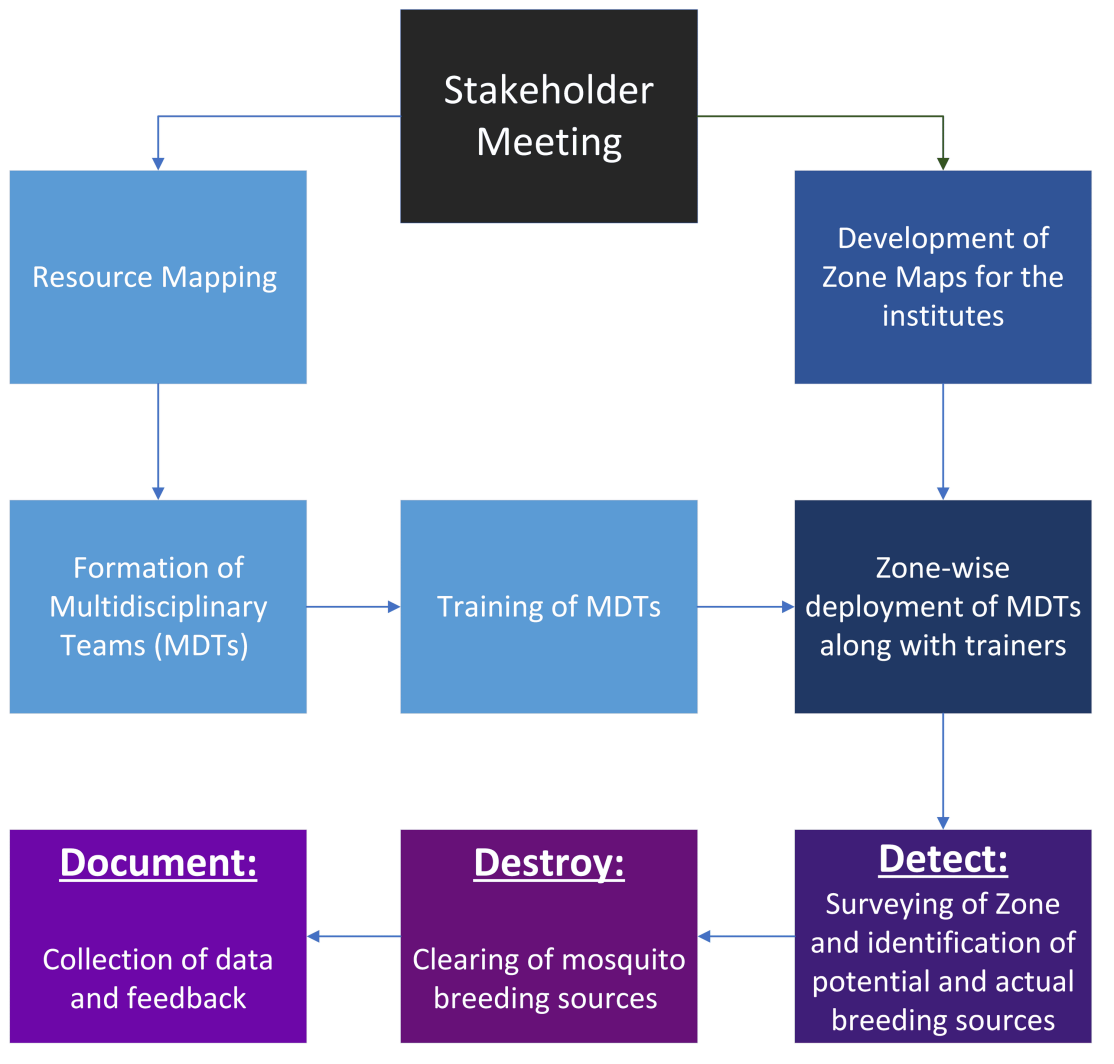
Action plan formulated following stakeholder engagement MDTs: multidisciplinary teams

Based on the discussions with the stakeholders, an activity plan was formulated as shown in Figure 2.

Cognition evaluation

The training program's effectiveness was evaluated through pre- and post-tests administered to all participating multidisciplinary teams (n=60). Overall knowledge and perception scores significantly improved after the training, with the mean score increasing from 6.79 (±1.16 SD) to 8.07 (± 0.89 SD) out of a total of 9 (paired t-test, p<0.001) (Table 1). Notably, the proportion of participant teams that understood the importance of weekly DSR activities rose significantly (McNemar's Chi-squared test, p=0.0005) from 38 (63.3%) to 52 (86.7%) teams (Table 1).

**Table 1:** Comparative summary of the respondents' answers to the cognition assessment questions *Significant

Serial number	Question	Pre-test	Post-test	Test statistic	p value
		Number (%)	Number (%)		
		N=60	N=60		
1	What could be the cause of dengue fever?			McNemar's chi-squared test	
	Mosquito-borne	52 (86.7)	57 (95)	1.78	0.182
	Waterborne	8 (13.3)	3 (5)		
	Foodborne	0 (0)	0 (0)		
	Airborne	0 (0)	0 (0)		
	Don't know	0 (0)	0 (0)		
2	The mosquito that causes dengue fever breeds in clean water.			McNemar's chi-squared test	
	Yes	38 (63.3)	54 (90)	14.22	<0.001*
	No	22 (36.7)	6 (10)		
3	Dengue disease can spread through direct contact, coughing, or sneezing.			McNemar's chi-squared test	
	Yes	8 (13.3)	3 (5)	3.57	0.044*
	No	52 (86.7)	57 (95)		
4	Do you feel that there's a chance you will get dengue in the next one year?			McNemar's chi-squared test	
	Yes	10 (16.7)	33 (55)	21.04	<0.001*
	No	50 (83.3)	27 (45)		
5	How often should you clean your surroundings to eliminate breeding sites?			McNemar's chi-squared test	
	Weekly/multiple times a week	38 (63.3)	52 (86.7)	12.07	0.0005*
	Fortnightly/monthly/3 monthly	22 (36.7)	8 (13.3)		
6	Mosquitoes that cause dengue fever bite mainly during.			McNemar's chi-squared test	
	Daytime	51 (85)	55 (91.7)	1.5	0.219
	Night	9 (15)	5 (8.3)		
7	Are you confident you can identify and clear mosquito breeding sites around you?			McNemar's chi-squared test	
	Yes	41 (68.3)	56 (93.3)	10.71	0.001*
	No	19 (31.7)	4 (6.7)		
8	Select all the usual breeding sites of mosquitoes that cause dengue fever.			Paired t-test	
	Open containers	56 (93.3)	60 (100)		
	Tyres	52 (86.7)	55 (91.7)		
	Coconut shell	50 (83.3)	57 (95)		
	Cement tanks	49 (81.7)	56 (93.3)		
	Roof gutters	45 (75)	55 (91.7)		
	Indoor plant vase	40 (66.7)	54 (90)		
	Tree holes/leaf axils	40 (66.7)	50 (83.3)		
	Refrigerator tray	37 (61.7)	48 (80)		
	Grinding stone	35 (58.3)	48 (80)		
	Mean score±standard deviation	6.7±2.6	8.1±1.7	-3.59	0.0006*
9	Select all symptoms of dengue fever that you know.			Paired t-test	
	Fever	60 (100)	60 (100)		
	Body pain	59 (98.3)	60 (100)		
	Headache	55 (91.7)	60 (100)		
	Pain behind the eyes	45 (75)	46 (76.7)		
	Nausea/vomiting	40 (66.7)	41 (68.3)		
	Mean score±standard deviation	4.7±1.0	5.2±1.0	-2.9	0.0045*
10	Select all the practices that can prevent or control the spread of dengue fever.			Paired t-test	
	Prevent water stagnation	60 (100)	59 (98.3)		
	Close water storage containers	58 (96.7)	59 (98.3)		
	Use mosquito nets at night	58 (96.7)	45 (75)		
	Use mosquito aerosol spray	54 (90)	56 (93.3)		
	Use mosquito repellent	50 (83.3)	57 (95)		
	Manage waste properly	47 (78.3)	55 (91.7)		
	Mean score±standard deviation	5.1±0.9	5.6±0.8	-3.68	0.0005*
				Paired t-test	
	Mean correct scores±standard deviation	6.79±1.16	8.07±0.89	-10.01	<0.001*

**Figure 3:**
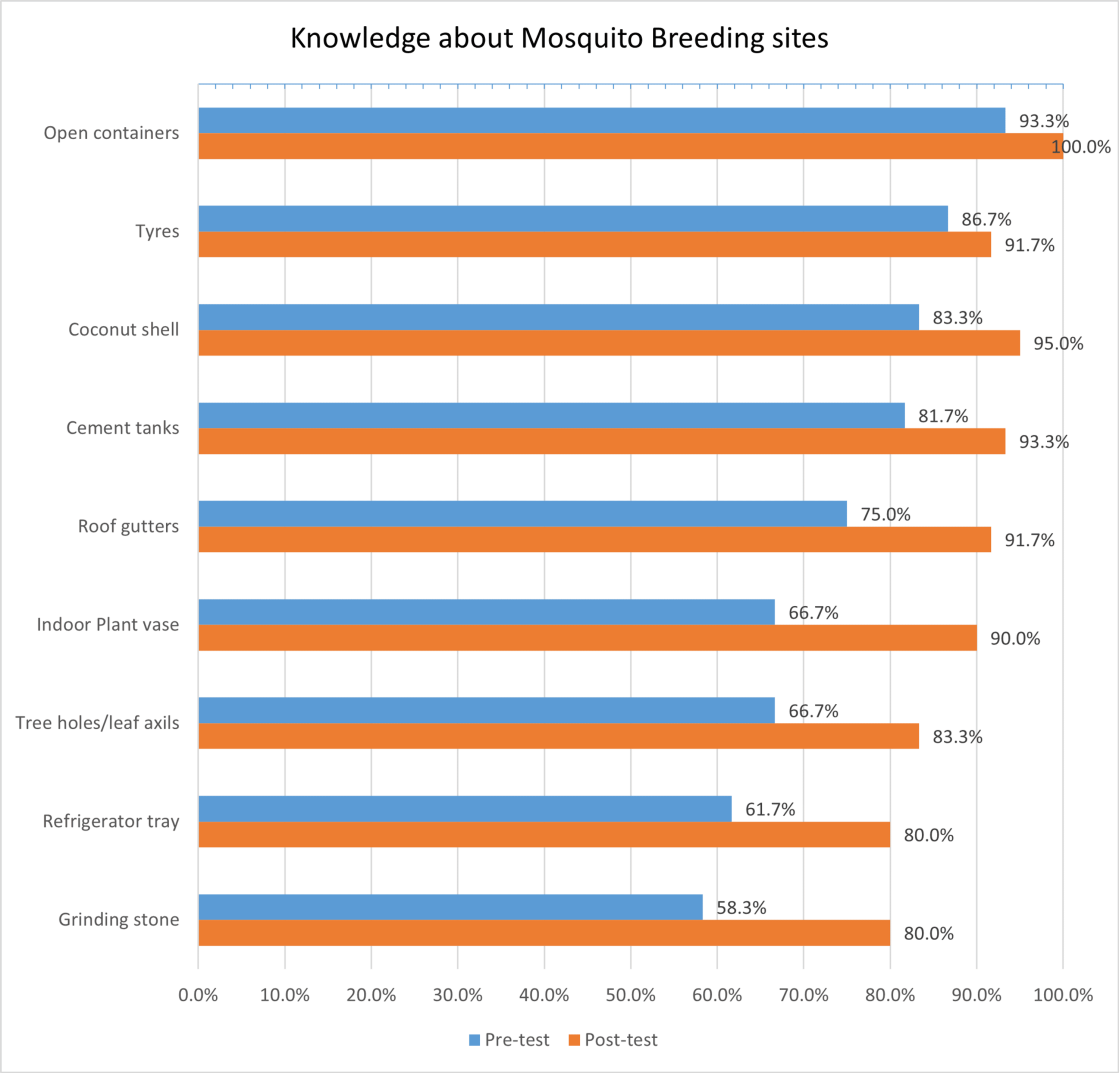
Bar graph illustrating the change in knowledge about mosquito breeding sites following training

While initial awareness about the cause (n=52, 86.7%), mode of disease transmission (n=52, 86.7%), and active biting hours of the vector (n=51, 85%) were satisfactory, understanding of the vector breeding sources was lacking. Before the training, only about two-thirds (n=38, 63.3%) of teams correctly identified clean water as a breeding source. However, following the training, most (n=54, 90%) recognized clean water as a breeding source, identifying common locations within their environment. The knowledge of indoor breeding sources, including plant vases and refrigerator trays, showed marked differences (Figure 3).

The training program also significantly impacted the participants' risk perception. Before the training, only 10 (16.67%) teams believed that they were likely to get dengue within a year. However, this proportion rose considerably to 55% following the training (McNemar's test, p<0.001). This suggests a heightened awareness of personal vulnerability. Additionally, the number of teams feeling confident in their ability to identify and eliminate mosquito breeding sites increased from 41 (68.3%) to 56 (93.3%) after the training session (McNemar's test, p<0.001). This demonstrates a considerable gain in confidence regarding vector control efforts, which was further assessed by capacity evaluation.

Capacity evaluation

Following the training, the multidisciplinary teams successfully conducted DSR activities under supervision. Ten days later, the MDTs independently carried out a second round of DSR activities with comparable results, demonstrating their ability to function effectively without external support.

During the initial supervised activity, the MDTs identified 118 containers, with 93 (78.8%) containing water (wet). Among these 93 wet containers, 37 (39.8%) contained immature stages (eggs, larvae, and pupae) of *Aedes *mosquitoes, the dengue vector. In the second, independent round, the MDTs identified 175 containers, of which 144 (82.3%) were wet. *Aedes *immatures were found in 64 (44%) of the 144 wet containers. These were confirmed by the supervising teams. Discarded plastic containers were the key breeding source, followed by flowerpots and iron or plastic drums. A detailed breakdown of the number of containers and mosquito breeding sites identified during both the DSR activities is shown in Table 2.

**Table 2:** Breeding containers identified during initial and follow-up DSR activities DSR: Dengue Source Reduction

Container types	Initial DSR activity			Follow-up DSR activity		
	Containers (C)	Wet containers (WC)	Containers with mosquito (immature forms) (number (%)) (N=WC)	Containers (C)	Wet containers (WC)	Containers with mosquito (immature forms) (number (%)) (N=WC)
Discarded plastic containers	36	32	17 (53.1)	34	32	16 (50)
Flowerpot/saucer under the flowerpot	31	10	6 (60)	38	19	10 (52.6)
Iron/plastic drums	6	6	3 (50)	20	19	8 (42.1)
Discarded bottles/cups/covers	9	9	1 (11.1)	17	16	4 (25.0)
Overhead plastic/cement tanks	11	11	5 (45.4)	11	11	1 (9)
Coconut shell	2	2	1 (50)	13	7	3 (42.9)
Discarded utensils	4	4	1 (25)	4	2	2 (100)
Discarded tyres	1	1	1 (100)	3	3	3 (100)
Tree hole/plant axils	1	1	0	5	5	1 (20)
Fridge tray/air conditioner drain	0	0	0	5	5	0
Water stagnation	0	0	0	7	7	5 (71.4)
Others	17	17	2 (11.8)	18	18	11 (61.1)
Total	118	93	37 (39.8)	175	144	64 (44.4)

Table 2 presents data from initial and follow-up Dengue Source Reduction (DSR) activities. Chi-squared test analysis revealed significant increases in total containers (118 to 175, p<0.001) and wet containers (93 to 144, p<0.001) between activities, indicating a more robust surveillance. It is to be noted that the follow-up activity did not have any supervisory team members guiding them. While it was not directly supervised, the teams submitted images of the breeding containers, which were checked by the supervisory team to verify. However, the proportion of wet containers with immature forms of mosquitos showed no significant change (39.8% to 44.4%, p=0.4767). This consistency in immature levels, despite increased container identification, suggests that the quality of the survey remained adequate and consistent across both activities, validating the reliability of the surveillance method. These findings show the improved capacity of the trained teams for conducting *Aedes* breeding source surveys.

The mosquito larvae and pupae collected during field activities were brought back to the Centre's laboratory facility and were allowed to emerge inside a mosquito cage. The emerged adults (172) from the second activity were identified at the species level. All collected samples belonged to the *Aedes* genus, with* Aedes aegypti*, the primary vector of dengue, being the most prevalent (n=105, 61.05%), followed by *Aedes albopictus *(n=67, 38.95%).

Feedback

Stakeholder and participants' feedback underscored the study's influence on the perceptions of staff and students and the challenges encountered during the activity. Notable challenges included concerns about sustainability, difficulties in accessing extensive unmanned areas such as construction sites, the continuous generation of waste from diverse sources, and the potential conflicts with academic schedules. Many stakeholders felt that the program could not be sustainable as the students would keep changing (n=3) or due to the staff and management losing interest in prolonged preventive activities (n=8). Some (n=4) were also apprehensive about checking the large areas of the medical college grounds that were not easily accessible due to construction (n=3) and vegetation (n=2) or were simply too large to check regularly (n=2). The stakeholders involved in the campus upkeep (n=3) also felt that due to the enormous amount of general waste that is generated on campus by students, staff, hostel residents, patients, and visitors, this activity would need to be done regularly. The academic faculty (n=6) mentioned that regular DSR activities could interfere with class schedules, and students may not be interested in continuing the activity without additional motivation or benefits. However, all the stakeholders (n=14) felt that the activity was important for the prevention of dengue on campus, and they opined that the activity should also be implemented in the communities where the medical college is located.

## Discussion

Dengue fever remains a continuous public health threat, necessitating multifaceted approaches to control the vector mosquitoes. This study explored a capacity-building strategy using interactive training and hands-on field activities, implemented within medical colleges. The findings underscore the transformative potential of such interventions, particularly in enhancing knowledge, risk perception, and practical vector control skills. Training medical students and engaging healthcare professionals who frequently interact with communities, has the potential for a dual impact.

The preliminary discussions held with the academic heads of the medical colleges emphasized the importance of stakeholder receptivity for the study's success. A self-study guide on stakeholder engagement by the Centre for Disease Control and Prevention (CDC) proved valuable in understanding how to involve stakeholders throughout the entire process, including the evaluation phase. The stakeholders are more likely to support and act on our recommendations if they are already a part of the process, without which any evaluation may get ignored or criticized [10]. Before designing the intervention, we assessed the campus layout, human resource structure, academic schedules, and student availability. This comprehensive approach allowed us to tailor action plans that would maximize participant engagement and receptiveness, ultimately leading to stronger support and action on our recommendations. The significance of stakeholder engagement in designing and implementing institutional initiatives is further emphasized by similar studies conducted globally [13,14].

Interactions with the professors and those in charge of various departments of the medical colleges including the campus upkeep department were instrumental in mapping the available resources and aligning the study with existing academic and campus activities. Forming multidisciplinary teams proved crucial. Involving students, faculty, and the housekeeping staff ensured a holistic vector control effort. Similarly, articles by various researchers have highlighted the importance of involving multidisciplinary teams in improving healthcare outcomes in diverse medical domains [15-17]. Dividing the campus into zones facilitated systematic coverage during field exercises.

The training program adopted a learner-centered approach with innovative hands-on elements. Live mosquito specimens (eggs, larvae, and pupae) were used for demonstrations, and participants received practical training during DSR activities. Additionally, training was delivered in both English and the local language (Tamil) to promote interaction and create an inclusive learning environment for all participants, aligning with best practices in capacity building [18,19]. Our approach aligns with the concept of systemic capacity building emphasized in the literature [19-21]. The framework proposed by FAIMER resonates with our study, highlighting the importance of identifying suitable participants, providing effective training with practical sessions, and guiding them on reporting and sustaining acquired knowledge [22]. The learners were enthusiastic about participating in the training and were interested in viewing the various life stages of mosquitoes up close. The post-test results displayed a learning level improvement, while the hands-on participation and independent screening of the breeding site showed a marked change in behavior, especially in subsequent screening exercises. The overall impact of the training was seen with the follow-up DSR activity identifying more breeding sites.

The Kirkpatrick Model facilitated a thorough evaluation of the training, highlighting strengths in participant engagement and learning, effective behavior change, and positive public health outcomes [12]. Our intervention resulted in a measurable improvement in participants' knowledge, risk perception, and confidence regarding dengue control. This included a better understanding of preventive measures and clinical symptoms. Significant improvements in participants' knowledge and behavior align with similar studies that have demonstrated the effectiveness of health training programs in raising awareness. For instance, previous research in vector control training has highlighted the utility of practical, learner-centered approaches for behavior change, which our findings support [23]. However, a notable addition to earlier studies is the significant increase in participants' risk perception (from 16.7% to 55%, p<0.001). This indicates that incorporating risk communication as part of the training can enhance the psychological readiness to adopt preventive practices. Enhanced confidence and risk perception are crucial achievements in the context of disease control, particularly for vector-borne diseases such as dengue [24].

A clear and consistent monitoring and reporting system proved valuable for both investigators and participants. It allowed for the assessment of DSR activity effectiveness and the identification of challenges. The feedback mechanism facilitated ongoing communication, enabling teams to share experiences, troubleshoot issues, and suggest improvements. The participants positively responded to the "Detect, Destroy, Document (3D)" strategy. This approach aligns with literature advocating for iterative feedback and adaptive implementation in public health interventions [19,20].

The study aligned with the guidelines of the National Center for Vector Borne Diseases Control (NCVBDC) for mosquito control in hospitals [25]. Discarded plastic containers, flowerpots, and disposable cups/lids were the most frequently identified breeding sites during both DSR activities. Our findings regarding the identification of key breeding sources, such as discarded containers and flowerpots, resonate with studies conducted in other regions, emphasizing the universal nature of these risk factors [26-28]. The improved ability of participants to identify these containers and the immature mosquito forms (confirmed as *Aedes *through adult emergence) highlights the effectiveness of the comprehensive training, which included live demonstrations and hands-on field practice under supervision.

While this study focused on dengue prevention, the capacity-building strategy has broader implications. The multidisciplinary team approach, combined with hands-on training and robust monitoring, could be adapted to other vector-borne diseases such as chikungunya, zika, and malaria. Tailoring the training modules to include disease-specific elements, such as the characteristics of *Anopheles* mosquitoes for malaria, could enable effective replication of this strategy in regions with varying epidemiological profiles.

Moreover, the integration of community participation alongside institutional efforts could enhance the scalability of this model [29]. By engaging medical students and faculty as change agents, this strategy could also address urban and peri-urban health challenges in similar resource-limited settings globally [30]. For instance, adapting the strategy to include sanitation drives and education on waterborne diseases could amplify its impact in areas affected by concurrent outbreaks of multiple diseases.

Future work should explore how variations in implementation (e.g., training duration and participant demographics) influence outcomes, thereby refining the model's application in public health training. While the study demonstrated short-term gains, further research is needed to assess the retention of knowledge and behavioral changes over time. Regular follow-ups and evaluations can provide deeper insights into sustaining the intervention's impact. The integration of this initiative into a broader public health framework including its applicability in schools, workplaces, or community centers could be explored to extend its reach. Incorporating digital tools such as mobile applications for monitoring DSR activities and sharing real-time feedback could enhance the efficiency and accuracy of interventions.

Strengths and limitations

The study offers valuable insights into building capacity for dengue control within medical colleges. A key strength lies in the innovative training plan developed through comprehensive stakeholder engagement. The multidisciplinary team trained for DSR activities effectively implemented the plan. The structured reporting system facilitated a successful repeat of the field activity during the follow-up phase. However, it is important to acknowledge the limitations. The study relies on data from a single follow-up evaluation, providing limited information on the program's long-term sustainability. Repeated follow-up data are crucial to ensure the sustainability and effectiveness of capacity-building and training programs. Additionally, the follow-up results rely on self-reported data by participants, lacking direct observation for verification. We also acknowledge that variations in local infrastructure, resources, and cultural context may impact the effectiveness of similar interventions in different settings. Future research could explore these variables, particularly how educational environments and local health infrastructure shape the adoption and sustainability of vector control practices especially when expanded to other settings.

Recommendations

To ensure long-term impact, this study highlights the importance of incorporating sustainability planning. This includes ongoing capacity-building efforts, sustained stakeholder engagement, fostering a sense of ownership among participants, and extending the study's influence beyond its initial implementation. The establishment of a dedicated Vector Control Unit within medical colleges, staffed and trained by interdisciplinary volunteers, is recommended. A systematic plan for risk communication and sensitization within the campus and the surrounding community is also crucial. Collaboration with student organizations, such as the National Service Scheme (NSS), could be explored, with student teams taking ownership of designated zones for DSR activities. Integrating weekly "dry day" activities (days with a focus on eliminating mosquito breeding sites) into the Competency-Based Medical Education (CBME) program could further solidify these practices. The use of the Family Adoption Programme under the new CBME guidelines for medical education to teach about dengue and source reduction to the adopted villages and families will also help in dissemination and community mobilization for dengue source reduction. Recognition and award programs could provide additional motivation for sustained participation. Regular monitoring visits and a robust monitoring and evaluation system are essential to ensure program effectiveness. Furthermore, collaborative efforts with the state's National Vector Borne Disease Control Programme (NVBDCP) could be undertaken to develop targeted awareness campaigns for both the college campus and the surrounding community.

## Conclusions

This study demonstrates that interactive, hands-on capacity-building programs in medical colleges can significantly improve knowledge, risk perception, and practical skills in vector control for dengue prevention. The training intervention led to measurable improvements, such as increased knowledge scores, heightened awareness of dengue risk, and enhanced understanding of weekly DSR activities. The trained multidisciplinary teams effectively identified and eliminated mosquito breeding sites, with their independent follow-up activity identifying more containers and maintaining consistency in the identification of mosquito immature forms. The incorporation of the "Detect, Destroy, Document" approach facilitated robust monitoring and evaluation, while stakeholder engagement ensured alignment with institutional priorities. The study highlights the role of medical colleges in spearheading sustainable dengue control measures, particularly through structured training programs and collaborative action plans. Integration of these findings into medical curricula and institutional frameworks has the potential to sustain these efforts and significantly impact community health outcomes.
